# Interferon-β Suppresses Murine Th1 Cell Function in the Absence of Antigen-Presenting Cells

**DOI:** 10.1371/journal.pone.0124802

**Published:** 2015-04-17

**Authors:** Nicolas Boivin, Joanie Baillargeon, Prenitha Mercy Ignatius Arokia Doss, Andrée-Pascale Roy, Manu Rangachari

**Affiliations:** 1 Department of Neuroscience, Centre de recherche du CHU de Québec—Université Laval, Québec QC, Canada G1V 4G2; 2 Graduate Programme in Microbiology and Immunology, Faculty of Medicine, Université Laval, Québec QC, Canada G1V 0A6; 3 Department of Molecular Medicine, Faculty of Medicine, Université Laval, Québec QC, Canada G1V 0A6; Escola Paulista de Medicina—UNIFESP, BRAZIL

## Abstract

Interferon (IFN)-β is a front-line therapy for the treatment of the relapsing-remitting form of multiple sclerosis. However, its immunosuppressive mechanism of function remains incompletely understood. While it has been proposed that IFN-β suppresses the function of inflammatory myelin antigen-reactive T cells by promoting the release of immunomodulatory cytokines such as IL-27 from antigen-presenting cells (APCs), its direct effects on inflammatory CD4^+^ Th1 cells are less clear. Here, we establish that IFN-β inhibits mouse IFN-γ^+^ Th1 cell function in the absence of APCs. CD4^+^ T cells express the type I interferon receptor, and IFN-β can suppress Th1 cell proliferation under APC-free stimulation conditions. IFN-β-treated myelin antigen-specific Th1 cells are impaired in their ability to induce severe experimental autoimmune encephalomyelitis (EAE) upon transfer to lymphocyte-deficient *Rag1^-/-^* mice. Polarized Th1 cells downregulate IFN-γ and IL-2, and upregulate the negative regulatory receptor Tim-3, when treated with IFN-β in the absence of APCs. Further, IFN-β treatment of Th1 cells upregulates phosphorylation of Stat1, and downregulates phosphorylation of Stat4. Our data indicate that IFN-γ-producing Th1 cells are directly responsive to IFN-β and point to a novel mechanism of IFN-β-mediated T cell suppression that is independent of APC-derived signals.

## Introduction

Multiple sclerosis (MS) is a progressive neurologic disease in which components of the nervous myelin sheath degenerate, leading to axonal loss and ultimately to neuronal dysfunction and disability. MS presents a significant burden to public health; for example, in Canada, it is estimated that 240 of every 100,000 people suffer from the disease [1]. While the etiology of MS is complex [2], a substantial body of evidence indicates that MS is primarily a T-lymphocyte-mediated autoimmune disorder in which myelin-reactive T cells cross the blood-brain barrier and direct an attack against central nervous system (CNS) myelin that is characterized by the infiltration of inflammatory neutrophils and macrophages. Immunohistochemical analysis of acute and recent MS lesions revealed extensive perivascular infiltration of T lymphocytes [3], and a multicenter genome-wide association study found that genes encoding T cell-related signaling molecules and cytokines were strikingly over-represented among MS-associated single nucleotide polymorphisms [4]. In addition, several currently available MS therapies, including glatiramer acetate [5] and interferon (IFN)-β [6], are thought to disrupt the inflammatory T cell response. The T-cell-mediated aspects of MS pathology are readily capitulated by the murine experimental autoimmune encephalomyelitis (EAE) model of CNS autoimmunity [7]. The evidence indicates that IFN-γ-producing CD4^+^ Th1 cells and interleukin (IL)-17-producing CD4^+^ Th17 cells [8,9] play a crucial role in the pathogenesis of human MS and murine EAE, and suggest that these inflammatory CD4^+^ T cell subsets co-operate to promote CNS autoimmunity.

IFN-β has emerged as a front line disease-modifying therapy for the relapsing-remitting form of MS that can reduce both the frequency of relapses as well as the formation of new lesions [10]. However, while IFN-β is known to exert its effects by modulating the inflammatory functions of T cells, its precise mechanism of function is not fully understood. It has been shown that IFN-β can modulate effector T cell function indirectly via its effects on antigen-presenting cells (APCs) such as macrophages and dendritic cells. Prinz et al. [11] demonstrated a crucial role for the type I interferon receptor on myeloid cells in suppressing EAE. Further, IFN-β induces secretion of the immunosuppressive cytokine IL-27 from APCs [12], resulting in the suppression of encephalitogenic Th17 cells [13]. However, it is less clear whether IFN-β acts directly on encephalitogenic T cells. Several studies have shown that T cells from IFN-β-treated MS patients [14–16] or EAE rodents [17,18] display defective inflammatory capacity. Nonetheless, impaired *ex vivo* function of T cells from IFN-β-treated subjects in these studies could reflect the indirect activity of IFN-β on APCs *in vivo*. Similarly, the majority of studies in which primary T cells are shown to be susceptible to IFN-β-dependent modulation *in vitro* are dependent on mixed leukocyte culture conditions in which IFN-β could potentially act on APCs as well as T cells [16,19–21]. Further, while a series of provocative studies showed that IFN-β can induce an immunomodulatory phenotype in Th17 cells [11,13,22–24], the effects of IFN-β on Th1 responses are incompletely understood, despite the fact that IFN-β can have differential effects on the regulation of Th1-driven versus Th17-driven CNS autoimmunity [18].

In this study, we show that IFN-β can suppress Th1 responses in the absence of APCs. Under these conditions, IFN-β profoundly inhibits Th1 cell proliferation as well as the ability of myelin antigen-specific 2D2 T cells to induce severe EAE. Differentiated and restimulated IFN-β-treated Th1 cells display an impaired ability to generate IFN-γ and IL-2, and these cells upregulate expression of the negative regulatory receptor T cell immunoglobulin and mucin domain-containing-3 (Tim-3). These data indicate that IFN-β can directly suppress Th1 responses, through mechanisms distinct from its ability to induce IL-27 production from APCs.

## Materials and Methods

### Ethics statement

This study was carried out in strict accordance with the guidelines set out by the Canadian Council on Animal Care. All breedings and experimental protocols were approved by the Animal Protection Committee of the Centre de recherche du CHU de Québec—Université Laval (CPA-CHUQ; protocol #13-070-2). All efforts were made to minimize suffering, following standard operating procedures mandated by the CPA-CHUQ. Mice were euthanized by exposure to carbon dioxide after first being anesthetized with isofluorane. Cervical dislocation was used as a secondary method of euthanasia.

### Animals and EAE induction

C57BL/6J, 2D2 and *Rag1* ^*-/-*^ mice were obtained from the Jackson Laboratories (Bar Harbor ME). *IFNAR1* ^*-/-*^ mice (Ifnar1^<tm1Agt>^) were obtained from the Mutant Mouse Regional Resource Centers (MMRRC). 2D2 mice [25] were maintained under our established breeding protocol at the CHU de Québec—Université Laval. Six (6) to 10 week old female mice were used in all experiments. For *in vivo* T cell transfer, stimulated cells were injected in cold PBS (Cellgro) intravenously, 5 x10^6^ cells per *Rag1* ^*-/-*^ recipient. Recipient animals received 200 ng of pertussis toxin (List Biological Labs) intraperitoneally on day 0 (d0) and d2 post-transfer. Animals were monitored daily for the development of EAE according to the following criteria: 0, no disease; 1, decreased tail tone; 2, hind limb weakness or partial paralysis; 3, complete hind limb paralysis; 4, front and hind limb paralysis; 5, moribund state [26]. Mice were sacrificed at the indicated timepoints.

### Reagents and cytokines

Flow cytometry monoclonal antibodies against mouse antigens (CD4, clone RM4-5; CD8a, clone 53–6.7; CD62L, clone MEL-14; IFN-γ, clone XMG1.2; IL-2, clone JES6-16E3; Ifnar1, clone MAR1-5A3; PD-1, clone RMP1-30; T-bet, clone 4B10) were obtained from Biolegend. Tim-3 monoclonal antibody (clone 5D12) was a gift from V. Kuchroo. Carboxyfluorescein succinimidyl ester (CFSE) was obtained from Biolegend. The following recombinant cytokines and antibodies were used for *in vitro* T cell cultures: mIFN-β (PBL InterferonSource, indicated concentrations), mIL-12 (R&D Biosystems, 10 ng mL^-1^), mIL-2 (Miltenyi, 5 ng mL^-1^), hTGF-β (Miltenyi, 3 ng mL^-1^), mIL-6 (Miltenyi, 20 ng mL^-1^), mIL-23 (R&D Biosystems, 20 ng mL^-1^), anti-mIL-4 (BioXCell; clone 11B11, 10 μg mL^-1^), anti-CD3 (eBioscience; Functional Grade Purified, clone 145-2C11, 2 μg mL^-1^), anti-CD28 (Biolegend; LEAF-purified, clone 37.51, 2 μg mL^-1^). Western blotting primary antibodies were obtained from BD Transduction Laboratories (Stat1, clone 42/Stat1, dilution 1:1000; Stat1 pY701, clone 14/P-STAT1, dilution 1:1000; Stat4 pY693, clone 42/Stat1, dilution 1:500), Cell Signaling (Stat4, clone C46B10, dilution 1:500) or Millipore (GAPDH, mAb374, dilution 1:1000). Anti-mouse or-rabbit secondary antibodies were obtained from Jackson Immunoresearch and were used at 1:10000.

### Flow cytometry

Staining for cell surface markers was carried out at 4°C (30 minutes). Prior to incubation with monoclonal antibodies against cell surface markers, cells were incubated for 10 minutes in the presence of Fc Block (BD Biosciences) to prevent non-specific antibody binding to cells. For analysis of intracellular cytokine expression, cells were first cultured for 4 hours in the presence of 50 ng mL^-1^ phorbol 12-myristate 13-acetate (Sigma-Aldrich), 1 μM ionomycin (Sigma-Aldrich) and GolgiStop (1 μL per mL culture; BD Biosciences). Cells were subsequently incubated with Fc Block and fluorescent cell surface marker antibodies and were then fixed and permeabilized using Fixation and Perm/Wash buffers (Biolegend). They were then stained with fluorescent antibodies against intracellular markers. For detection of T-bet, staining was completed using True-Nuclear Transcription Factor Buffer Set (Biolegend) according to the manufacturer’s instructions. For analysis of cell proliferation, cells were labeled with 5 μM CFSE for 5 minutes at room temperature prior to culture. Flow cytometry data were collected using an LSRII flow cytometer (BD Biosciences) and were analyzed using FlowJo software (TreeStar). Dead cells were excluded from data analysis on the basis of positivity for Viability Dye (eBioscience). Gates were set on the basis of fluorescence minus one (FMO) controls or isotype control staining (for Tim-3).

### T cell isolation and culture

Single cell suspensions were obtained from lymph nodes and spleens, and were enriched for CD4^+^ and CD8^+^ T cell populations using monoclonal antibody-labeled magnetic beads and columns (Miltenyi). To obtain purified T cell naïve or effector populations, enriched CD4^+^ and CD8^+^ T cells were subsequently labeled with fluorescent monoclonal antibodies against CD62L, and either CD4 or CD8 (all Biolegend), and were sorted into CD4^+^CD62L^hi^ (naïve), CD8^+^CD62L^hi^, CD4^+^CD62L^lo^ (effector) or CD8^+^CD62L^lo^ populations using a FACSAria digital cell sorter (Becton Dickinson). For Th differentiation, culture plates were coated with 2 μg ml^-1^ each of anti-CD3 and anti-CD28 in PBS, overnight at 4°C. Plates were washed with PBS, and T cell populations were added at a concentration of 10^6^ cells ml^-1^ under Th1 (IL-12+anti-IL-4) or Th17 (TGF-β+IL-6) conditions. Cells were cultured in DMEM (Life Technologies), supplemented as described [27]. After 2 days, cells were transferred to uncoated plates with IL-2 (Th1) or IL-23 (Th17) for an additional 3 days. For polarization of cells into differentiated effectors, cells were restimulated with platebound antibodies and the appropriate cytokines/blocking antibodies 5 days after the initial stimulation. APCs were generated by irradiating (2000 rad) red blood cell-lysed CD4^-^ or CD8^-^ eluate from magnetic cell separation (as described above). T cells were cultured with APCs at a 1:5 ratio where required.

### CNS cell isolation

Mice were euthanized as described above and were perfused with cold PBS administered through the left cardiac ventricle. Brains and spinal cords were dissected from the skull and spinal column, respectively. CNS tissue were homogenized using a PTFE Tissue Grinder (VWR) and were incubated for 30 minutes at 37°C in homogenization solution (HBSS containing 4 ng ml^-1^ liberase and 25 ng ml^-1^ DNase). Homogenate were filtered through a 70 μm cell strainer, resuspended in 35% Percoll (GE Healthcare) and centrifuged. Mononuclear cells were collected, washed, and prepared for flow cytometric analysis.

### Western blotting

Prior to collection, cultured cells were incubated for 30 minutes at 37°C with 10 uM bpVphen (Sigma) in order to inhibit endogenous phosphatases. Cells were then washed twice in PBS and lysed in cell lysis buffer (RIPA buffer, Boston BioProducts; 10% SDS; 100mM Na_3_VO_4_; 100 mM PMSF; 1X Halt Protease/Phosphatase Inhibitor Cocktail, ThermoScientific). Boiled and clarified lysates were quantitated using DC Protein Assay (Bio-Rad) prior to addition of Laemmli buffer containing 5% β-mercaptoethanol. Equivalent amounts of protein were loaded onto Criterion TGX precast gels (Bio-Rad), which were subsequently transferred to PVDF (Bio-Rad). Membrane were blocked in 5% milk-Tris buffered saline containing 0.05% Tween-20 (TBST), and were incubated with primary antibodies overnight at 4°C at the concentrations indicated in “Reagents and cytokines”. Membranes were washed and incubated for 1 hour with the relevant HRP-conjugated anti-mouse or-rabbit secondary antibody (anti-mouse in all cases except Stat4). Washed membranes were incubated briefly with ECL Reagent (GE Healthcare) and subsequently developed on BIOMAX MR Film (Carestream). For detection of multiple targets on the same membrane, RestorePLUS buffer (Thermo Scientific) was used to strip membranes.

### Statistical analyses

For comparisons of two sets of normally distributed data, two-tailed, unpaired, Student's *t*-test was used. For analysis of differential onset of disease between EAE groups, the slopes of the disease onset curve were obtained by performing linear regression analysis [26,28,29]. Statistical analyses were performed using Prism (GraphPad).

## Results

**Fig 1:**
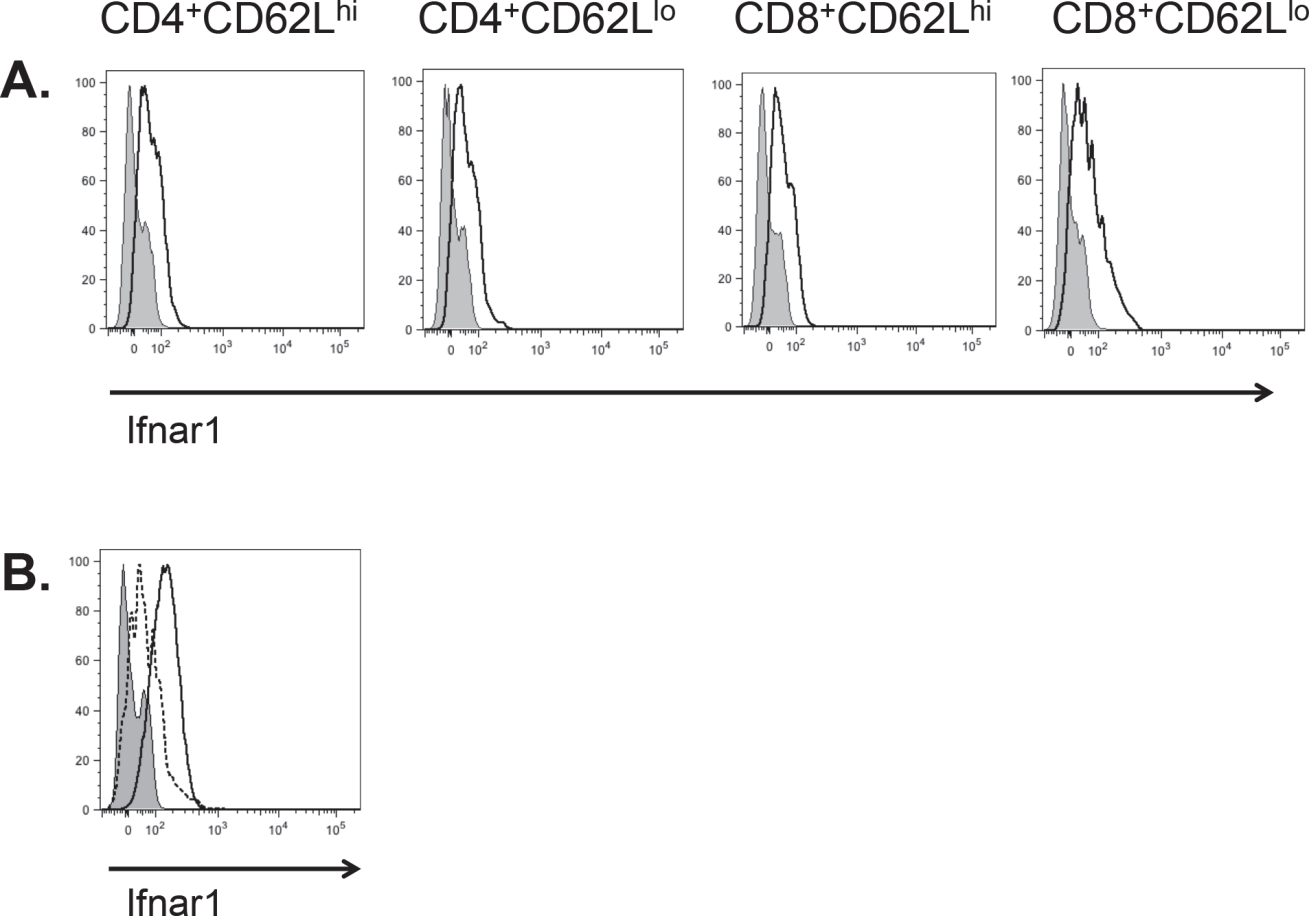
Th1 cells express the type I interferon receptor. A. Naïve (CD62L^hi^) and effector (CD62L^lo^) CD4^+^ and CD8^+^ C57BL/6J T cells were assessed for surface expression of Ifnar1 by flow cytometry. Open histogram, Ifnar1; shaded histogram, fluorescence minus one (FMO) control. B. Th1 and Th17 cells were cultured and assessed for surface expression of Ifnar1 after 5 days. Solid line with open histogram, Th1; Dotted line with open histogram, Th17; shaded histogram, FMO control. Data representative of one of three experiments.

IFN-β signals through the IFN-α/β receptor (IFN-α/β-R), which consists of receptor chains 1 and 2. We first sought to confirm that CD4^+^ T cells express the IFN-αR complex. As expression of IFN-α/β-R on CD8^+^ T cells is critical for the ability of mice to clear vaccinia virus and lymphocytic choriomeningitis virus infection [30], we also assessed CD8^+^ T cell expression of IFN-α/β-R in parallel. We found that CD4^+^ T cells from unmanipulated mice express Ifnar1 on their surface at levels comparable to those seen in CD8^+^ T cells. Both naïve (CD62L^hi^) and effector (CD62L^lo^) T cells express IFN-α/β-R, suggesting that T cells are potentially responsive to IFN-β at multiple stages of differentiation (Fig 1A). As both Th1 and Th17 responses are implicated in the pathogenesis of MS and EAE [8,9], we wanted to assess the expression of Ifnar1 on Th1 and Th17 cells. We found that *in vitro* differentiated Th1 cells expressed higher levels of Ifnar1 than their Th17 counterparts (Fig 1B).

**Fig 2:**
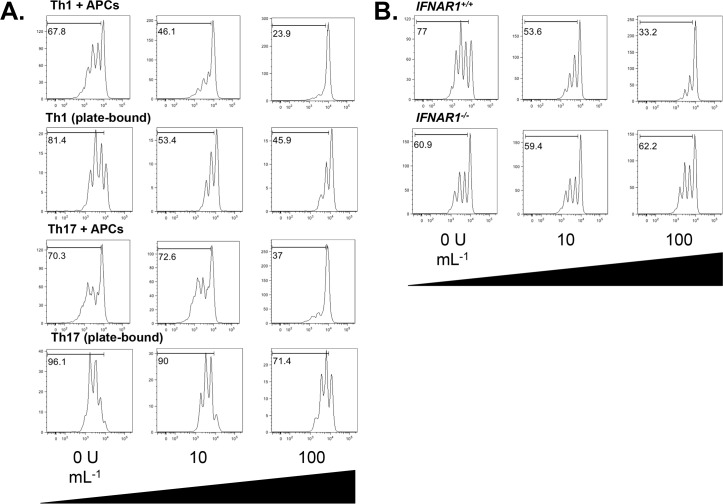
IFN-β suppresses Th1 cell proliferation. A. WT CD4^+^CD62L^hi^ T cells were labeled with CFSE and stimulated for 5 days with either soluble anti-CD3 plus irradiated splenocytes (APCs), or with plate-bound anti-CD3+anti-CD28, under Th1 or Th17 conditions, with the indicated concentrations of IFN-β. CFSE dilution was assessed by flow cytometry. Data representative of three experiments. B. WT or *IFNAR1-/-* CD4^+^CD62L^hi^ T cells were labeled with CFSE and stimulated with plate-bound anti-CD3+anti-CD28 under Th1 conditions with the indicated concentrations of IFN-β for 5 days. CFSE dilution was assessed by flow cytometry. Data representative of two experiments. Gates represent the percentage of cells that underwent at least one division.

Having determined that Th1 cells express IFN-α/β-R, we next wanted to determine whether they could respond to treatment with IFN-β. Previous studies had analyzed the effects of IFN-β function on Th1 cells while using B cells [19], fibroblasts [20,22], irradiated splenocytes [14,31], purified dendritic cells [32] or whole blood leukocytes [33] as APCs. Here, we wished to examine whether IFN-β could modulate Th1 cell function in the absence of APCs. Thus, we stimulated naïve CD62L^hi^ CD4^+^ T cells with immobilized anti-CD3 and anti-CD28 monoclonal antibodies (plate-bound stimulation). This protocol mimics physiologic activation of T cells via concomitant stimulation through both their T cell receptor and the costimulatory receptor CD28. Using this plate-bound approach allows us to isolate the effects of IFN-β on T cells in the absence of APC-derived cues. To generate Th1 cells, we stimulated naïve CD62L^hi^ CD4^+^ T cells *in vitro* in the presence of the Th1 driving cytokine IL-12. In addition, we used a blocking antibody to IL-4 in order to suppress Th2 cell differentiation. As expected, IFN-β suppressed the proliferation of Th1 cells cultured with APCs, as measured by CFSE dilution. Interestingly, IFN-β also suppressed the proliferation of Th1 cells stimulated by anti-CD3+anti-CD28, indicating that its antiproliferative effects do not require APC-derived signals (Fig 2A). This effect was dependent on the expression of IFN-α/β-R, as IFN-β had little effect on the proliferation of *IFNAR1* ^*-/-*^ Th1 cells stimulated under APC-free conditions (Fig 2B). As we found that IFN-α/β-R is more highly expressed on Th1 cells than Th17 cells (Fig 1B), we wanted to ascertain whether IFN-β differentially regulates the proliferation of Th1 versus Th17 cells. Thus, we generated Th17 cells by stimulating CD62L^hi^CD4^+^ T cells with anti-CD3 and anti-CD28 in the presence of TGF-β and IL-6 [34], and in the presence (10 U mL^-1^, 100 U mL^-1^) or absence of IFN-β. We found that while IFN-β could suppress Th17 cell proliferation, it only did so at a dose of 100 U mL^-1^. By contrast, as little as 10 U ml^-1^ IFN-β inhibited Th1 cell proliferation (Fig 2A). Taken together, these data indicate that IFN-β can suppress the proliferation of Th1 and Th17 cells in the absence of APCs and that this effect is more pronounced in Th1 cells.

**Fig 3:**
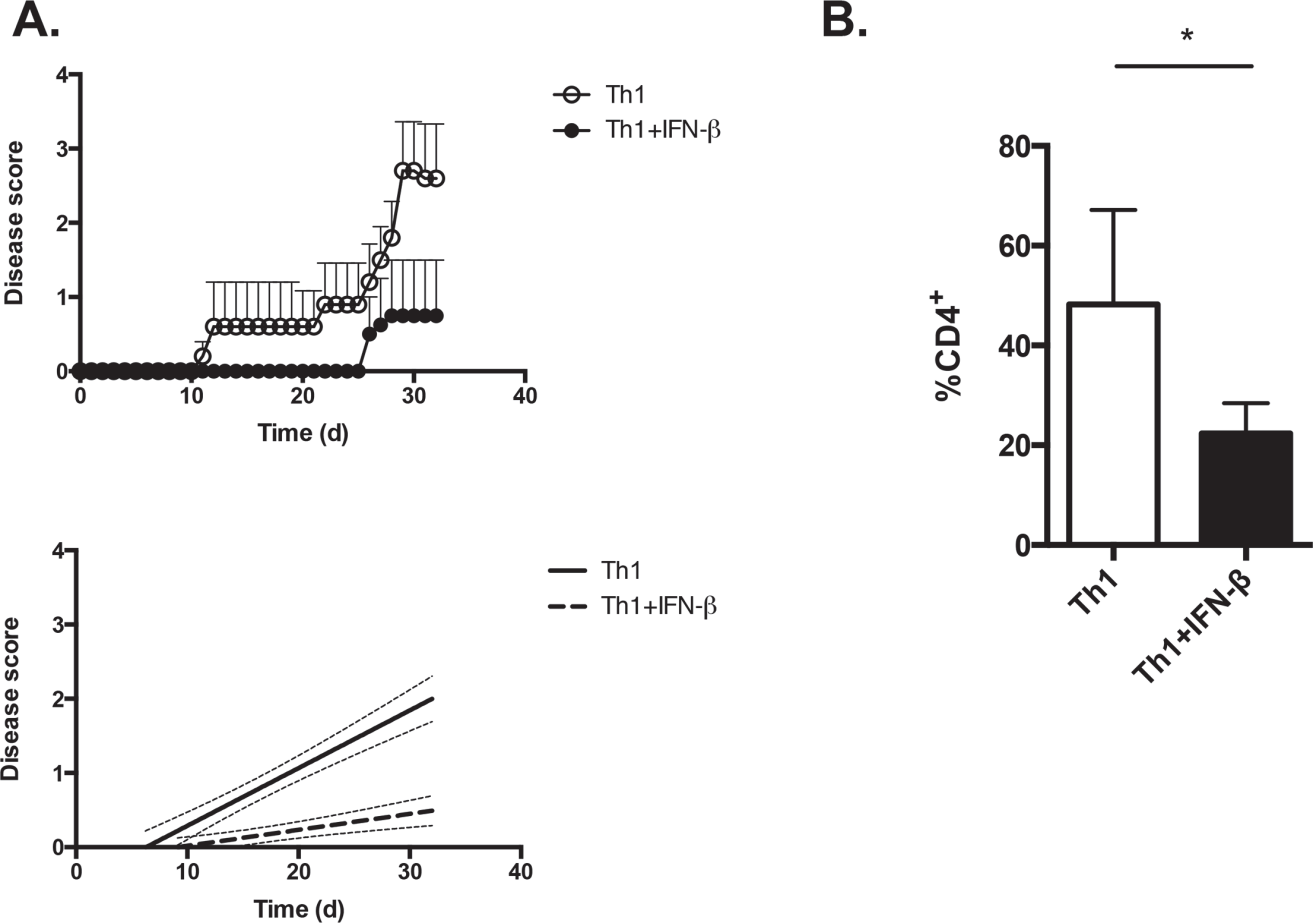
IFN-β suppresses the encephalitogenic potential of Th1 cells. CD4^+^CD62L^hi^ T cells were isolated from female 2D2 mouse spleens and lymph nodes, and were stimulated with plate-bound anti-CD3+anti-CD28 under Th1 conditions, in the presence or absence of 100 U mL^-1^ of IFN-β, for 5 days. They were then transferred to female, 6-week old, *Rag1-/-* mice (5x10^6^ cells/mouse). Recipient mice were monitored for clinical signs of EAE. n = 5 Th1, n = 4 Th1+IFN-β. Right graph, linear regression curves of the disease courses. The slopes are significantly different between the disease courses (p<0.0006). B. Brains and spinal cords were isolated from mice in (A) at d32 and single mononuclear cell suspensions were obtained. The frequency of CNS-infiltrating CD4^+^ cells was assessed by flow cytometry. * p<0.05, two-tailed Student’s *t* test.

It was recently suggested that IFN-β selectively inhibits Th1-mediated CNS autoimmunity and antigen-specific Th1 responses in mice [18]. In this study, mice that had received myelin antigen-specific effector Th1 cells were subsequently injected with recombinant IFN-β over the course of the disease. Thus, it remains unclear whether IFN-β can directly inhibit myelin-specific Th1 cell responses, or whether the effects of IFN-β on Th1 cells in EAE are the indirect result of IFN-β acting on bystander APCs. To isolate the direct effects of IFN-β on the encephalitogenic potential of myelin-reactive T cells, we exploited a recently described model of myelin antigen-specific T cell transfer in which myelin oligodendrocyte glycoprotein (MOG)_[35–55]_-specific transgenic CD4^+^ T cells (2D2 cells) are stimulated *in vitro* under defined differentiation conditions and are then transferred to non-immunized recipients to induce EAE [35]. We sorted CD4^+^CD62L^hi^ T cells from 2D2 mice [25] and stimulated them with anti-CD3+anti-CD28 under Th1 differentiation conditions, in the presence or absence of IFN-β. After 5 days of culture under Th1 conditions, cells were transferred to lymphocyte-deficient *Rag1* ^*-/-*^ recipients. These mice were then monitored for the development of EAE. We found that recipients of IFN-β-treated 2D2 Th1 cells developed EAE of lessened severity when compared to mice that received 2D2 Th1 cells that had not been cultured with IFN-β (Fig 3A). Further, the percentage of CNS-infiltrating CD4^+^ T cells was lower in IFN-β-treated 2D2 recipients than in control 2D2 recipients (Fig 3B). Taken together, these data indicate that exposure to IFN-β during the priming phase of T cell stimulation can diminish the capacity of Th1 cells to infiltrate the CNS and induce EAE.

**Fig 4:**
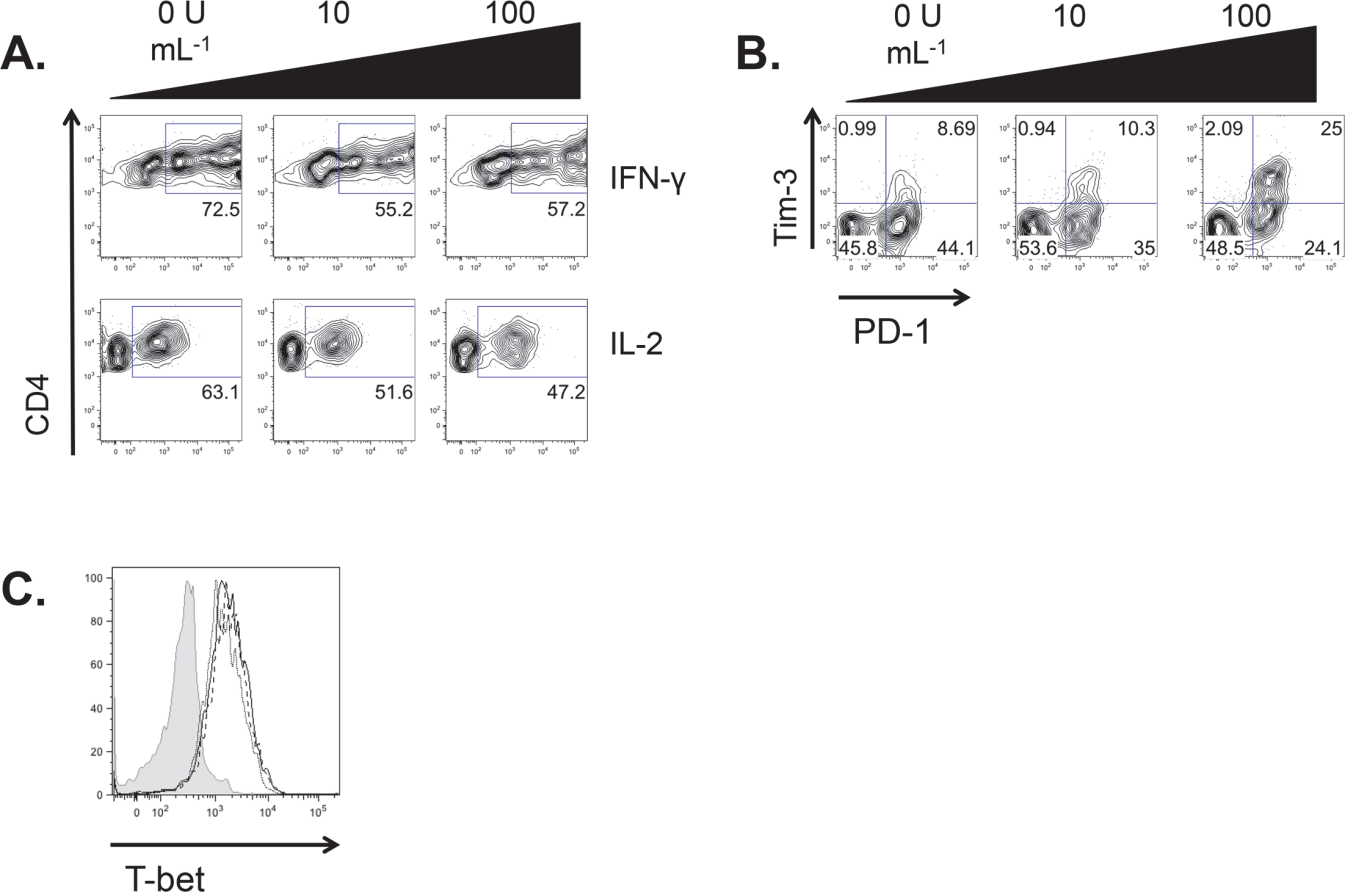
IFN-β suppresses cytokine secretion from Th1 cells. A. CD4^+^CD62L^hi^ T cells were sorted from the spleens and lymph nodes of C57BL6/J mice and were stimulated with plate-bound anti-CD3+anti-CD28 under Th1 conditions, with the indicated concentrations of IFN-β, for 5 days. Cells were restimulated for 48 hours under Th1 conditions with the same concentration of IFN-β as at the initial stimulation. A. Generation of IFN-γ and IL-2 was assessed by intracellular cytokine staining and flow cytometry. Representative of three experiments. B. Expression of Tim-3 and PD-1 were assessed by flow cytometry. Representative of three experiments. C. Expression of T-bet was assessed by flow cytometry. Representative data from one of three mice assessed individually. Solid line with open histogram, Th1; dashed line with open histogram, Th1 + 10 U mL^-1^ IFN-β; dotted line with open histogram, Th1 + 100 U mL^-1^ IFN-β; shaded histogram, FMO control. Data in all panels gated on CD4^+^ cells.

Given our finding that IFN-β could suppress Th1 cell-mediated EAE through an APC-independent mechanism, we wished to more closely examine the effects of IFN-β on effector Th1 cell function. We generated Th1 cells from CD4^+^CD62L^hi^ naïve progenitors by plate-bound stimulation in the presence or absence of IFN-β. After 5 days, we restimulated the cells with plate-bound antibodies for an additional 48 hours in the continued presence or absence of IFN-β. We found that IFN-β reduced the percentage of polarized effector Th1 cells that generated IFN-γ and IL-2 as measured by flow cytometry (Fig 4A), demonstrating that IFN-β can downregulate an effector Th1 cell phenotype.

Tim-3 is a cell surface receptor specifically upregulated on differentiated effector Th1 cells that negatively regulates their function [36,37]. Its expression can also mark a subset of exhausted, dysfunctional, T cells that previously responded vigorously to antigen, thus correlating with the loss of production of IFN-γ [26,38,39] and IL-2 [26,38]. As we found that IFN-β could downregulate expression of IFN-γ and IL-2 from Th1 cells, we therefore examined whether it could enhance their expression of Tim-3 in parallel. Upon restimulation with plate-bound anti-CD3 and anti-CD28, 100 U mL^-1^ of IFN-β induced a substantial increase in Tim-3 expression on Th1 cells (Fig 4B). These data indicate that IFN-β can induce the expression of the negative regulatory receptor Tim-3 on effector Th1 cells in the absence of APCs. Interestingly, the majority of Tim3^+^ Th1 cells were also positive for the negative regulatory receptor programmed cell death-1 (PD-1); however, IFN-β did not increase total expression of PD-1 (Fig 4B). Further, IFN-β had little effect on the expression of T-bet (Fig 4C), the master regulator of Th1 responses [40] that can also regulate the expression of Tim-3 [41].

**Fig 5:**
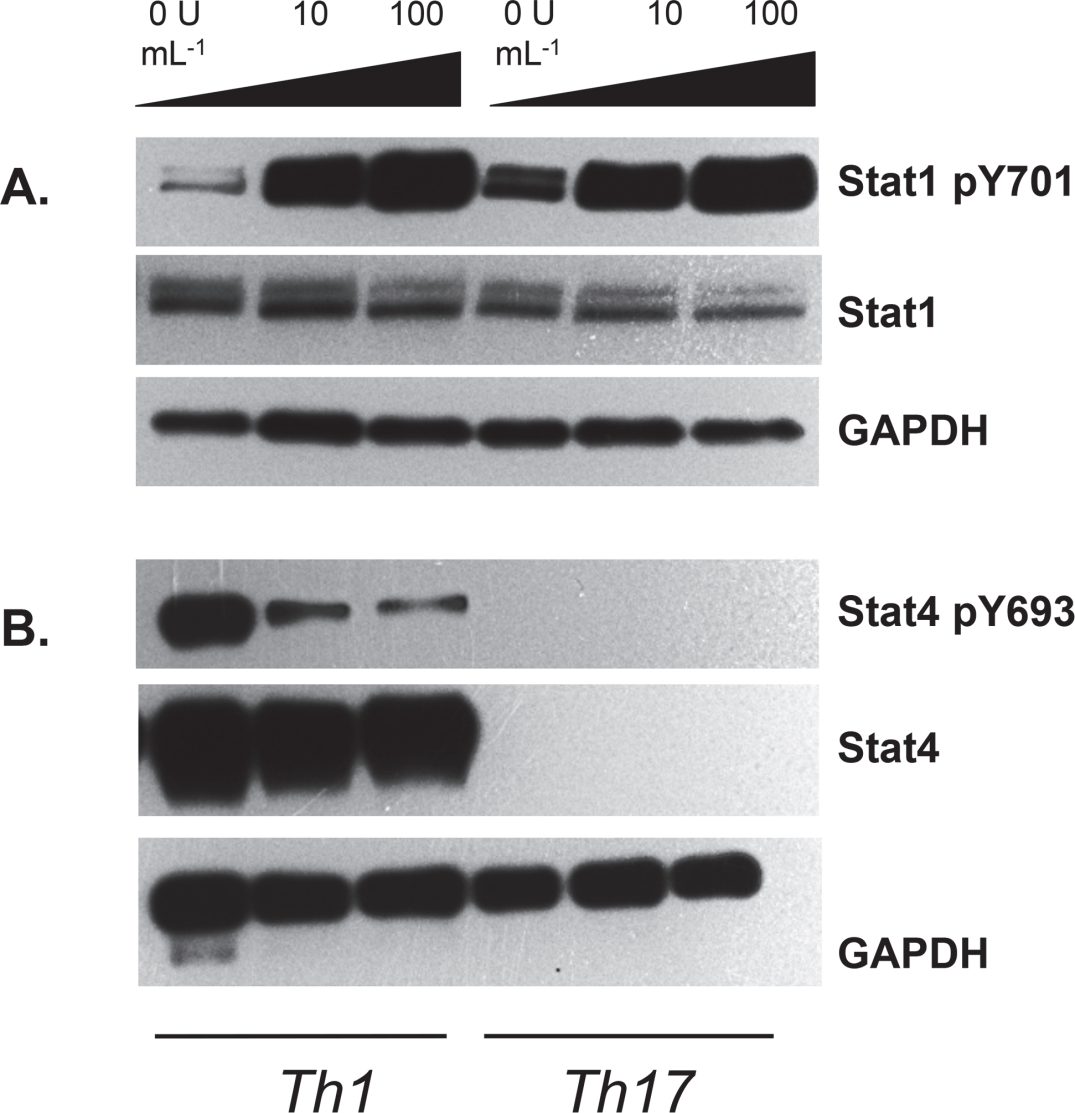
IFN-β regulates Stat1 and Stat4 expression on Th cells in the absence of APCs. CD4^+^CD62L^hi^CD25^-^ T cells were sorted from the spleens and lymph nodes of C57BL6/J mice. They were stimulated with plate-bound anti-CD3+anti-CD28 under Th1 or Th17 conditions, with the indicated concentrations of IFN-β, for 48 hours, and cell lysates were generated. A. Expression of Stat1 and pStat1(Y701) were assessed by Western blot. Representative of two experiments. B. Expression of Stat4 and pStat4(Y693) were assessed by Western blot. Representative of three experiments. GAPDH, loading control.

Stat1 and Stat4 are transcription factors that are crucial to initial steps in Th1 cell differentiation. Stat1 is phosphorylated upon T cell exposure to IFN-γ, while Stat4 activity is induced by IL-12 [42]. As our data indicate that IFN-β can modulate Th1 cell function *in vitro* and *in vivo*, we wanted to examine its effects on Stat1 and Stat4 activity in Th1 cells. To our surprise, we found that IFN-β strongly induced Stat1 phosphorylation at tyrosine residue 701 (Y701) in Th1 and Th17 cells. (Fig 5A). It was previously demonstrated that Th17 differentiation conditions can induce Stat1 [43]. By contrast, phosphorylation of Stat4 at Y693 in Th1 cells was markedly reduced by IFN-β (Fig 5B). These data suggest that while IFN-β can enhance sensitivity of Th1 cells to IFN-γ, it downmodulates their response to the key Th1 differentiation cytokine IL-12.

## Discussion

IFN-β was approved as a disease-modifying treatment for relapsing-remitting MS in 1993 and it remains a front-line therapy [44]. It reduces the frequency of disease exacerbations [45,46] as well as the accumulation of inflammatory MS lesions in the CNS [47]. However, it is not universally effective, and it has been suggested that nearly half of MS patients are non-responsive to IFN-β [48]. It is most effective in the early stages of disease [49]; when applied upon the advent of a clinically isolated demyelinating event, evidence suggests that IFN-β can delay the onset of clinically defined MS [50]. However, it has modest efficacy in modulating the progressive phase of MS [47]. It has recently been suggested that IFN-β treatment may be of limited use in patients whose serum cytokine profile skews towards one characterized by elevated IL-17F, and that IFN-β may in fact exacerbate disease symptoms in these patients [18,51]. Others have found that patients who respond poorly to IFN-β therapy have a different pattern of type I interferon-regulated gene expression in whole blood relative to patients who respond well [52]. On the whole, despite having been used in the clinic for over 20 years, the mechanism of function of IFN-β in the context of MS remains incompletely defined [53]. Better understanding of these mechanisms could help us to a) define subsets of patients who would be likely to respond well to this therapy, b) identify biomarkers that could predict responsiveness versus non-responsiveness to therapy and c) characterize novel signaling pathways that could be the target of multi-reagent therapy.

It has been suggested that IFN-β enhances the stability of the blood brain barrier by both enhancing endothelial tight junctions [54] and by downregulating the expression of matrix metalloproteinases [45]. However, a consensus has emerged suggesting that the primary function of IFN-β in modulating MS is to repress the function of myelin antigen-specific T cells [55]. IFN-β treatment of T cells from both MS patients and healthy controls decreased both their proliferative capacity as well as their ability to secrete IFN-γ [14], and the frequency of IFN-γ^+^ and IL-2^+^ peripheral blood mononuclear cells (PBMCs) was reduced in MS patients undergoing IFN-β treatment as compared to therapy naïve patients [56]. Further, addition of IFN-β to CD4^+^ T cell cultures increased their production of the immunomodulatory cytokine IL-10 [19].

However, while essential in defining an immunomodulatory role for IFN-β, these *ex vivo* and *in vitro* analyses did not specifically address whether the suppressive effects of IFN-β on T cell function are due to its direct activity on IFN-α/β-R-expressing T cells, or whether they are the indirect result of its effects on APCs. Indeed, IFN-β therapy modulates the expression of APC costimulatory molecules on PBMCs from MS patients [57] and downregulates dendritic cell and monocyte expression of IL-12 [32,58], the critical Th1 differentiation factor. IFN-β upregulates the secretion of the immunomodulatory cytokine IL-27 from human dendritic cells, and the inability of certain patients to upregulate IL-27 in response to IFN-β has been suggested as an explanation for their lack of responsiveness to this therapy [12]. Thus, despite a prominent report demonstrating that IFN-β profoundly diminishes CNS-specific Th1 cell reactivity in EAE [18], it was important to establish the effects of IFN-β on Th1 cells in the absence of APCs.

Here, we demonstrate that IFN-α/β-R is expressed on *ex vivo* isolated CD4^+^ T cells at similar levels as seen on CD8^+^ T cells; as type I interferons induce the proliferation of memory CD8^+^ T cells [59] and are essential for the *in vivo* clearance of viral infections [30], this suggests that IFN-α/β-R plays a physiological role on CD4^+^ T cells. Indeed, we find that its expression is upregulated under Th1 differentiation conditions. We therefore considered the possibility that in addition to its activity on APCs, IFN-β can have direct effects on Th cells that may in part explain its immunoregulatory activity. We find that even in the absence of APCs, IFN-β potently suppresses Th1 cell proliferation. Further, it inhibits the generation of IFN-γ and IL-2 from restimulated Th1 cells.

Deonarain et al. [60] previously dissected the function of IFN-β in the immune system development by studying *IFN-β* ^*-/-*^ mice. Consistent with our findings, they found that IFN-β-deficient T cells displayed enhanced proliferation when stimulated with plate-bound anti-CD3 and anti-CD28. Curiously, however, they also found that *IFN-β* ^*-/-*^ CD4^+^ and CD8^+^ T cells generate less IFN-γ, IL-2 and TNF-α than wildtype counterparts. There are several possible explanations for the discrepancies observed between these observations and our own. First, *IFN-β* ^*-/-*^ mice lack expression of the cytokine in all cell types, and the authors observed profound effects of IFN-β deficiency on granulocyte-macrophage precursors as well as bone marrow-derived macrophages [60]. Thus, it is possible that some of the phenotypes observed in *IFN-β* ^*-/-*^ T cells *ex vivo* are the indirect result of alterations in APC function under homeostatic conditions. Second, T cells in this study were cultured under neutral conditions as opposed to Th1 conditions. Here, we find that differentiated, plate-bound antibody-stimulated Th1 cells are specifically sensitive to the effects of IFN-β, suggesting that IFN-β synergizes with IL-12-dependent pathways in order to optimally suppress effector T cell function. Indeed, we find that IFN-β diminishes phosphorylation of the IL-12 signal transducer Stat4 in Th1 cells. The requirement for IL-12 in mediating the inhibitory effects of IFN-β may be an important area for further investigation.

Several studies have shown a role for endogenous IFN-β in suppressing Th17 responses in the context of EAE. Guo et al. [13] elegantly demonstrated that APCs produce IL-27 in response to IFN-β, in a manner dependent on the adaptor molecule TRIF, and that this mechanism was crucial to constraining Th17-dependent EAE. Similarly, Shinohara et al. [24] found that type I interferons induce IL-27 production from dendritic cells in an intracellular osteopontin-dependent manner, and that this resulted in blunted Th17-mediated EAE. Intriguingly, other studies have demonstrated that exogenous IFN-β suppresses Th17 responses under APC-free conditions [22], while *IFN-β* ^*-/-*^ Th17 cells produce enhanced amounts of IL-17 relative to wildtype counterparts [23]. Here, we find that while high doses of IFN-β can suppress the proliferation of Th17 cells stimulated in the absence of accessory cells, this effect is less pronounced than that seen on Th1 cells. Thus, IFN-β may play a role in globally suppressing CD4^+^ T cell functions under inflammatory conditions. However, while its effects on Th17 cells may be chiefly the indirect result of APC conditioning, we show that IFN-β can directly suppress the function of Th1 cells.

Using a Cre-Lox approach, Prinz and colleagues [11] reported that deletion of IFNAR1 in lymphocytes does not regulate the incidence or severity of EAE. Rather, they found that IFNAR1 expression on myeloid cells is a critical parameter in modulating EAE severity. While it represented an important advance, this study differed from ours in several ways. First, its focus was on the role of endogenous type I interferons in the control of *in vivo* inflammation. Thus, it did not rule out an immunomodulatory effect of exogenous IFN-β on lymphocyte function, which would more closely resemble the therapeutic use of IFN-β in the treatment of MS. Second, the authors induced disease in IFNAR1-deficient mice by active immunization with MOG_[35–55]_ in complete Freund’s adjuvant (CFA). EAE induction using CFA results in a mixed Th1/Th17 response in which Th17 may predominate [61,62]. We have demonstrated that Th17 cell proliferation is suppressed by IFN-β only at high doses. Loss of IFN-β signaling on myelin-reactive Th17 cells may therefore not affect their encephalitogenic potential, thus explaining the limited effect of IFNAR1 deficiency on the severity of T cell-mediated EAE induced by active immunization in the presence of CFA.

In an important recent study, Axtell and colleagues [18] isolated splenocytes from MOG_[35–55]_-immunized mice, restimulated them *in vitro* with MOG_[35–55]_ under either Th1- or Th17-skewing conditions, and transferred the cells to naïve recipients. These host mice were then treated with IFN-β, or with vehicle, over the course of the disease. They found that while IFN-β exacerbated EAE in Th17 cell recipients, it substantially ameliorated signs of disease in Th1 recipients. Further, T cells isolated from the spinal cords of IFN-β-treated Th1 cell recipients at the end of the experiment produced less IFN-γ than vehicle-treated Th1 recipients. They thus concluded that IFN-β specifically modulates Th1-, but not Th17-driven, EAE. However, as the treatment regimen involved systemic administration of IFN-β to adoptive transfer recipients, this study did not isolate the direct effects of IFN-β on Th1 cells alone. Here, we show that treatment of myelin antigen-specific Th1 cells with IFN-β *ex vivo* reduces the severity of EAE upon *in vivo* transfer. Thus, IFN-β may be able to suppress Th1 responses through two distinct mechanisms; one that signals directly through type I interferon receptor complexes expressed on CD4^+^ T cells, and one that indirectly promotes IL-10 secretion via the induction of IL-27 from APCs. We also have evidence that *ex vivo* treatment of myelin-specific Th17 cells with IFN-β can exacerbate their ability to induce EAE (not shown), in concordance with the observations of Axtell [18] who found that restimulation of *in vivo*-generated myelin-specific Th17 cells in the presence of IFN-β could increase their encephalitogenic potential. While we find that the antiproliferative effects of IFN-β are less pronounced on Th17 cells than on Th1 cells, it is possible that IFN-β can preferentially drive the differentiation of a pathogenic Th17 cell population. This presents a potential avenue for future research.

Intriguingly, we find that IFN-β upregulates the expression of Tim-3 on Th1 cells. Tim-3 is a Th1 cell-specific negative regulatory cell surface receptor [36,37] that has recently been identified as a functional marker of T cell exhaustion [26,38,39,63,64]. Exhausted T cells arise as a result of antigenic persistence during chronic viral infections such as HIV-1 and HCV as well in cases of cancer. They lose cytotoxic and inflammatory function in a hierarchical and progressive manner. IL-2 and IFNγ secretion are among the effector functions that are lost from exhausted cells [65]. We find that polarized Th1 cells treated with IFN-β in the absence of APCs generate less IL-2 and IFNγ than untreated Th1 controls, and that these IFN-β-treated cells substantially upregulate Tim-3. Further, while total expression of the exhaustion marker PD-1 [66] is not increased on IFN-β-treated Th1 cells, the majority of Tim-3^+^ cells generated under these conditions are also doubly positive for PD-1. This double-positive phenotype is thought to define a population of severely exhausted T cells with highly compromised effector functions [38,64]. Tim-3^+^ exhausted T cells have been identified in the context of HIV-1 [39], HCV [67], and tumors [38,63]. CD4^+^ T cells from MS patients undergoing IFN-β therapy express higher levels of Tim-3 than cells from therapy naïve patients [16,68]. Further, while Tim-3-mediated suppression of IFN-γ was impaired in CD4^+^ T cells from MS patients, it was restored by exogenous IFN-β [16]. In the future, it will be interesting to examine the idea that by inducing T cell exhaustion to self-antigen, possibly through treatment with IFN-β, one can convert an inflammatory autoimmune T cell response to a exhausted, self-limiting one. Further, IFN-β can induce the expansion of a programmed cell death 1 ligand 1 (PD-L1)-positive regulatory T cell population *in vivo*, and this phenomenon relies on expression of IFNAR1 on CD4^+^ T cells [69]. The role of IFN-β signaling in promoting regulatory T cell generation thus also merits further investigation.

We observe differential effects of IFN-β on the regulation of Stat1 and Stat4 in plate-bound stimulated Th1 cells. IFN-β reduced phosphorylation of Stat4 at a residue (Y693) whose phosphorylation is IL-12-dependent [70]. Importantly, Stat4 deficiency prevents the development of severe EAE [71]. Perhaps more surprisingly, we find that IFN-β can increase Stat1 phosphorylation. Stat1 activity is strongly induced by IFN-γ [42], but IL-12-dependent T cell proliferation and IFN-γ production are Stat1-independent [72]. Further Stat1-deficient mice develop highly severe EAE upon active immunization, as well as spontaneous EAE when crossed onto the 2D2 background [73]. Our data suggest that the modulation of effector Th1 cell function by IFN-β *in vitro* and *in vivo* is not caused by a downregulation of initial Th1 differentiation but rather by reducing Th1 cell sensitivity to IL-12-mediated signals. We found that the reduction in IFN-γ observed from IFN-β-treated Th1 cells was not paralleled by diminished expression of the Th1 transcription factor T-bet. However, T-bet is not strictly required for the expression of IFN-γ [74]. Rather, it may promote Th1 differentiation by repressing the Th2 differentiation factor IL-4 [74,75], which we block in our Th1 cultures. Intriguingly, it was recently shown that IL-27 can strongly induce Tim-3 expression in Th1 cells and that this mechanism partially requires T-bet [76]. These findings suggest that IFN-β may promote Tim-3 expression through both APC- and T-bet-dependent, and APC- and T-bet-independent, pathways.

While IFN-β remains a front-line immunomodulatory therapy for the treatment of MS, a substantial proportion of RRMS patients are non-responders [48,77]. Thus, it is crucial that we distinguish between APC-dependent and-independent effects of IFN-β on T cell function so as to better understand its mechanism of action. Our data indicate that IFN-β suppresses the function of Th1 cells in an APC-independent manner, at least in part by upregulating their expression of inhibitory T cell molecules such as Tim-3. Thus, the APC-independent inhibitory functions of IFN-β may complement its ability to suppress T cell function via APC-mediated signals. Further studies on the T cell-intrinsic immunomodulatory functions of IFN-β could help us to identify patient populations that can best respond to this therapy. They would also potentially permit us to uncover novel molecular therapeutic targets and to eventually design more effective reagents for the treatment of MS.
